# Combination of immune-checkpoint inhibitors and targeted therapies for melanoma therapy: The more, the better?

**DOI:** 10.1007/s10555-023-10097-z

**Published:** 2023-04-06

**Authors:** Maximilian Haist, Henner Stege, Michael Kuske, Julia Bauer, Annika Klumpp, Stephan Grabbe, Matthias Bros

**Affiliations:** 1 Department of Dermatology, University Medical Center Mainz, Langenbeckstraße 1, 55131 Mainz, Germany; 2 Department of Pathology, Stanford University School of Medicine, Stanford, CA 94305 USA; 3 Department of Microbiology & Immunology, Stanford University School of Medicine, Stanford, CA 94305 USA

**Keywords:** BRAF/MEK inhibitors, Immune-checkpoint inhibitors, Triple combination therapy, Sequential therapy, Metastatic melanoma; synergistic effects

## Abstract

The approval of immune-checkpoint inhibitors (CPI) and mitogen activated protein kinase inhibitors (MAPKi) in recent years significantly improved the treatment management and survival of patients with advanced malignant melanoma. CPI aim to counter-act receptor-mediated inhibitory effects of tumor cells and immunomodulatory cell types on effector T cells, whereas MAPKi are intended to inhibit tumor cell survival. In agreement with these complementary modes of action preclinical data indicated that the combined application of CPI and MAPKi or their optimal sequencing might provide additional clinical benefit. In this review the rationale and preclinical evidence that support the combined application of MAPKi and CPI either in concurrent or consecutive regimens are presented. Further, we will discuss the results from clinical trials investigating the sequential or combined application of MAPKi and CPI for advanced melanoma patients and their implications for clinical practice. Finally, we outline mechanisms of MAPKi and CPI cross-resistance which limit the efficacy of currently available treatments, as well as combination regimens.

## Introduction

Malignant melanoma is among the most aggressive solid tumors of the skin that displayed a fast-growing incidence in the last decades [1, 2]. Once melanoma has spread, it becomes life-threatening and until 2010 only few treatment options had been available for metastatic melanoma [1]. In the last decade, however, increased biological understanding of tumor-mediated immune evasion mechanisms substantially improved the treatment landscape of advanced melanoma [3]. In particular, the development of small molecule inhibitors that target the mitogen-activated protein kinase (MAPK) pathway, and checkpoint-modulating agents substantially improved both response and survival of patients with advanced melanoma [4].

The MAPK/extracellular-signal-regulated kinase (ERK) pathway has been identified as a critical signaling cascade in melanoma pathogenesis [5]. 40–50% of patients initially present with a mutation in the v-raf murine sarcoma viral oncogene homolog B (BRAF) kinase at position 600 (BRAF^V600^) [6, 7] resulting in the constitutive activation of the ERK pathway that promotes cell proliferation and inhibits apoptosis, thus driving tumorigenesis [6]. Single-agent BRAF inhibitors (BRAFi), including vemurafenib and dabrafenib, yielded initial response rates of 50% for patients with metastatic melanoma with a substantial prolongation of progression-free survival (PFS) to 7–9 months as compared to the standard chemotherapeutic agent dacarbazine [8, 9]. However, these tumor responses were typically short-lived due to reactivation of the MAPK pathway resulting in secondary resistance [10]. To this end, pharmacological inhibitors that target wild-type MAPK/ERK Kinase (MEK) downstream of BRAF were developed [11]. By now, combination therapies comprising dual application of BRAFi and MEK inhibitors (MEKi) constitute the standard regimen for metastatic BRAF^V600^ mutant melanoma, that achieve high tumor response rates of 75%, delay MAPK-driven acquired resistance with an increased PFS of 11–15 months and 5-year survival reaching 40% [12–15]. Meanwhile, BRAFi/MEKi showed a good safety and tolerability profile with approximately 20% of patients developing serious adverse events (AE) that include elevated liver enzymes (10%), elevated creatine phosphokinase (7%), but also retinopathy (12%), cardiomyopathy (8%) and QT-interval prolongations (3–4%).

Due to the rapid responses of MAPKi that are regularly observed within days of treatment initiation regardless of tumor burden and location of metastasis, combined BRAFi/MEKi therapy is particularly beneficial for symptomatic melanoma patients with rapidly progressing tumors [16]. Also, MAPKi may evoke tumor responses in case of melanoma brain metastases (MBM). In particular, dabrafenib + trametinib (DT) has shown intracranial response rates of up to 55% [17]. Nevertheless, secondary resistance and a relatively short duration of response with only 19% of patients being progression-free after 5 years remain major challenges for BRAF/MEKi therapies [15, 18].

The immunogenic profile of melanoma also contributed to the success of immune-checkpoint inhibitors (CPI). These monoclonal antibodies (mAb) exert activity through blockade of the programmed cell death-protein (PD)-1/PD-ligand 1 (PD-L1) axis or the cytotoxic T lymphocyte antigen 4 (CTLA-4)/CD80 and CD86 axis that blunts inhibitory T cell signaling and allows for the restoration of exhausted T cells [19]. Initial studies with the CTLA-4 blocking mAb ipilimumab (IPI) demonstrated a significantly prolonged treatment response in patients with metastatic melanoma and increased 3-year survival rates to 20% [20]. However, these studies also reported a range of dose-dependent inflammatory side effects for IPI, that affected the gastrointestinal system, liver, endocrine organs and the skin, occurring in up to 56% of patients [20, 21]. By contrast, PD-1 blocking agents (nivolumab and pembrolizumab) were associated with a more favorable toxicity profile with serious AE occurring in 17–21% of patients, while yielding superior efficacy both in terms of response rates (44% vs. 19%) and PFS (6.9 months vs. 2.9 months) as compared to IPI monotherapy [22–26]. Finally, the pivotal Checkmate-067 trial demonstrated that the combination of nivolumab (Nivo) and IPI (termed dual checkpoint blockade), was superior in terms of response (59% vs. 44%) and survival (11.5 months vs. 6.9 months) as compared to both either Nivo or IPI monotherapy, increasing the 5-year PFS to 38% and 5-year overall survival (OS) to over 50% for patients with metastatic melanoma [25, 26]. Notably, CPI also exerted activity in patients with BRAF-mutated melanoma and IPI plus Nivo combination even showed higher efficacy rates in this subgroup of patients as compared to melanoma patients with wild-type BRAF [25]. However, combination therapy was associated with higher rates of grade 3 or 4 serious AE that occurred in up to 56% of patients and 30% of patients had to discontinue combination therapy due to AE [25]. Therefore, both CPI monotherapy and combination therapy are considered the standard of care at this time [1]. More recently, the combination of Nivo and the lymphocyte activation gene (LAG-)3 targeting mAb relatlimab showed a more favorable toxicity profile with 19% of patients developing serious AE, while treatment efficacy was comparable to IPI + Nivo therapy [27].

Despite the significant progress of MAPKi and CPI therapies, there still is an unmet need to enhance treatment efficacy given the high rate of primary treatment resistance to CPI in approximately 50% of melanoma patients and secondary acquired resistance that is most commonly observed during treatment with MAPKi but has also been observed in 20–30% of CPI-treated patients. Importantly, upon tumor progression metastatic melanoma often displays a multidrug resistance phenotype that is less responsive to both MAPKi and CPI [28]. Therefore, it remains a major issue to identify treatment regimens that provide more durable responses in a first-line setting to avoid tumor relapse, as well as novel targets to overcome multidrug resistance upon tumor progression.

**Fig. 1:**
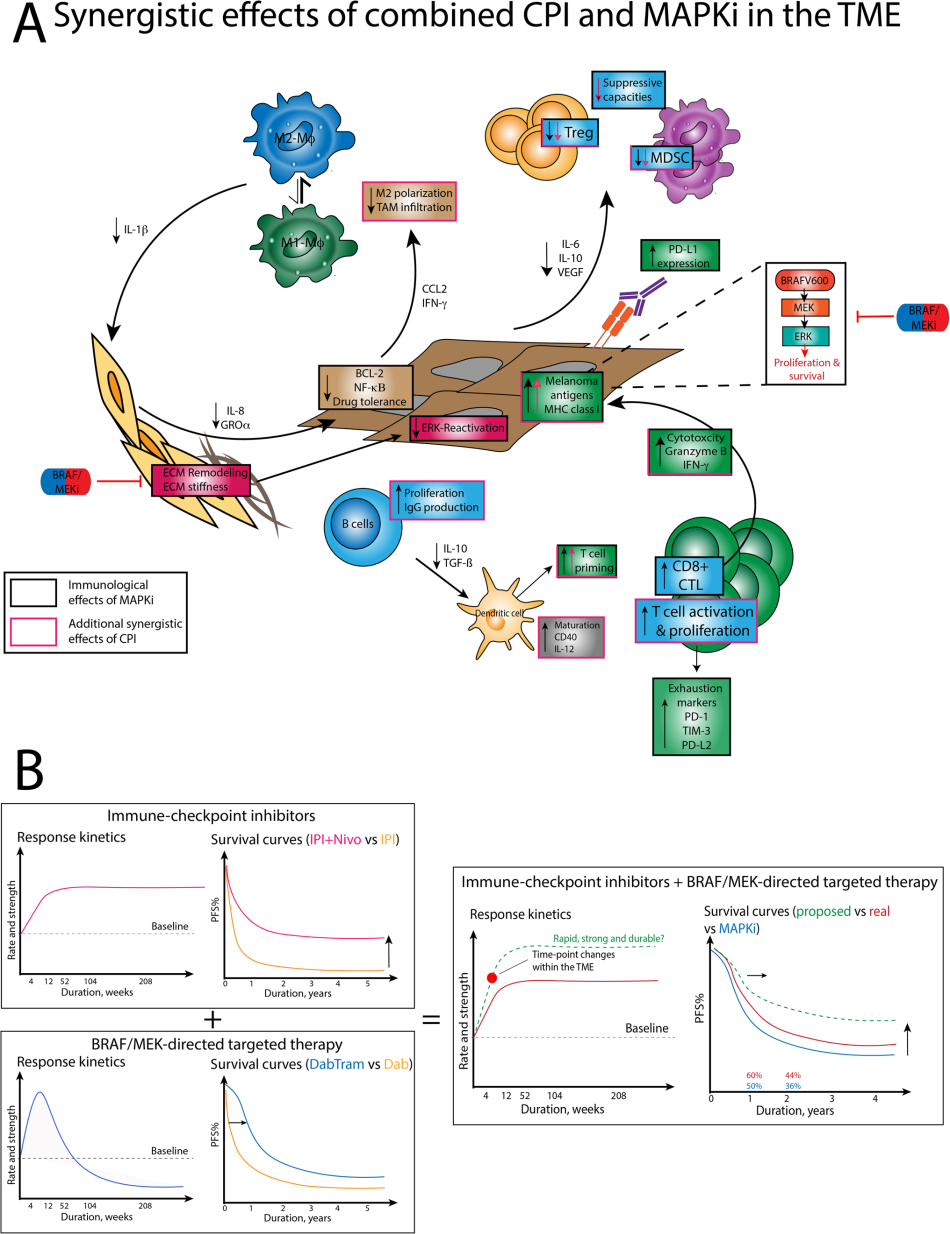
Mechanistic basis of the immunomodulatory effects of MAPKi and their synergy with CPI on the TME (A), as well as their individual and combined clinical response kinetics (B). Observations from preclinical studies reported that during the first 4 weeks MAPKi induce favorable changes within the TME that include a stronger infiltration by CD8 T cells, a reduced number of Treg and MDSC, as well as an enhanced priming capacity of antigen-presenting dendritic cells. The clinical response kinetics of MAPKi are visualized in the shape of Kaplan–Meier curves with early survival advantages of MAPKi becoming less pronounced over time and reaching a plateau at approximately 15%. By contrast, the addition of CPI, that have a slower onset of response, prolongs the initial rapid response induced by MAPKi and thus Kaplan Meier curves are characterized by less steep initial slopes but longer and higher plateauing tales at approximately 40%. However, those proposed kinetics did not entirely translate into the results of clinical trials that showed less pronounced benefits from this triple combination regimen. Abbreviations: IPI = ipilimumab; nivolumab = Nivo; DabTram = dabrafenib + trametinib; MAPKi = mitogen activated pathway kinase inhibitors; Dab = dabrafenib; TME = tumor microenvironment; ECM = extracellular matrix; Treg = regulatory T cells; MDSC = myeloid-derived suppressor cells; M1 Mϕ = M1 macrophages; M2 Mϕ = M2 macrophages; CTL = cytotoxic T lymphocytes; TAM = tumor-associated macrophages; PFS = progression-free survival

Evidence from preclinical studies and patient biopsies suggest that MAPKi have immune-modulating properties resulting in enhanced anti-tumor immune responses and a reversal of immune-evasive mechanisms within the tumor microenvironment (TME) [29–33]. Due to the synergistic immunological effects of CPI and MAPKi and their complementary clinical characteristics (see Fig. 1), it has been proposed that their combination might enhance overall treatment efficacy and overcome the primary CPI resistance that allows for more durable tumor responses with long-term PFS as assessed in ongoing clinical trials.

In this review, the biological rationale for the application of triple combination regimens, as well as the current knowledge from clinical trials on the safety and efficacy of triple combination regimens with MAPKi and CPI are discussed. Further, this review presents the potential of sequential application of MAPKi and CPI to enhance treatment efficacy. Last, we will give insights into mechanisms driving MAPKi and CPI cross-resistance that may be overcome by novel targeted therapies.

## Biological rationale

### Immunomodulatory effects of MAPK-inhibitors to augment CPI therapy

In addition to the direct anti-proliferative effects of MAPKi on melanoma cells, both BRAFi and MEKi exert off-target effects on immune cells, which in case of BRAFi are in large part mediated via paradoxical ERK activation in wild-type cells, whereas MEKi inhibit ERK signaling in all cells [6, 34]. In general, BRAF-mutated melanomas present with an immune-evasive phenotype characterized by the accumulation of immunomodulatory cytokines (i.e., IL-6 and IL-10) [35] and immunosuppressive cell types such as regulatory T cells (Treg) [36, 37] and myeloid-derived suppressor cells (MDSC) [38] within the TME. Moreover, tumor antigen presentation is attenuated via downregulation of MHC class I molecules and inhibition of antigen presenting cells (APC) [39, 40], thereby limiting the induction of effector T cells and T cell-mediated killing of tumor cells. BRAF/MEKi may in parts reverse the hostile microenvironment of BRAF-mutant melanomas [41] (see Fig. 1A). In particular, it has been observed in both preclinical and translation studies, that inhibition of BRAF and MEK enhanced MHC-I expression by tumor cells [42], the presentation of melanoma associated neo-antigens, including glycoprotein 100 (gp100) and melanoma antigen recognized by T cells (MART-1), [33, 43]. Also MAPKi resulted in a stronger influx of tumor infiltration lymphocytes (TIL) and a higher ratio of cytotoxic T cells (CTL) to Treg [30, 44]. In line, inhibition of ERK signaling attenuated recruitment of MDSC and Treg, as well as the production of immunosuppressive cytokines, including IL-6, IL-10 and vascular endothelial growth factor (VEGF), resulting in an overall cytokine shift towards an interferon-γ driven tumor micromilieu [29, 30, 45]. In addition, Frederick and co-workers reported increased levels of granzyme B and perforin by tumor-infiltrating CTL about 10–14 days after the onset of BRAF/MEKi therapy [33]. While these favorable modulations of the TME have largely been investigated for BRAF-mutant melanoma patients, it has also been observed that paradoxical MAPK/ERK activation has direct effects on T cells, NK cells and macrophages in wild-type BRAF non-tumor cells. Further, the administration of MEKi has been associated with favorable anti-tumor responses due to increased T cell proliferation capacities [46, 47].

Notably, translational studies observed a reversal of these favorable microenvironmental changes at the time of tumor progression during BRAF/MEKi treatment, as reflected by the upregulation of PD-L1 [48, 49], a reduction of CD8^+^ T cell/Treg ratio [33] and an increase of T cell exhaustion markers indicative of impaired T cell effector activities [28]. While the upregulation of PD-L1 during early BRAFi therapy may favor CPI response [50], acquired resistance to MAPKi during long-term BRAF/MEKi therapy driven by reactivation of the MAPK pathway in these tumors was found to promote an immune-evasive TME, that also mediated cross-resistance to CPI [28]. More importantly, it was also reported that long-term BRAF/MEKi therapy impaired antigen-presentation and effector T cell responses irrespective of secondary acquired MAPK-resistance, thus favoring the evolution of melanoma variants cross-resistant to MAPKi and CPI therapy [51, 52]. As a result of early preclinical studies, a biological rationale has emerged that supports the application of combination therapies of MAPKi with CPI to harness the early immune-stimulatory effects of BRAF/MEKi to overcome primary CPI-resistance, while avoiding the long-term emergence of an immunosuppressive TME that favors cross-resistance to CPI.

### Preclinical evidence on synergistic effects of MAPKi and CPI therapy

The prospect of combining MAPKi with CPI to enhance anti-tumor immunity for the treatment of metastatic melanoma has been proposed soon after their approval [53, 54] based on the encouraging results of a number of pre-clinical studies that reported synergistic effects of concomitant or consecutive application of MAPKi and CPI [55]. In the following we will summarize these preclinical data both for the concurrent administration of MAPKi and CPI as well as their sequential application.

Initial data from Cooper and co-workers demonstrated that the combination of anti-PD-1 or PD-L1 mAb with BRAFi slowed tumor growth, increased the number and activity of TIL, and accordingly prolonged survival in a BRAF^V600E^ phosphatase and tensin homolog (PTEN)^−/−^ syngeneic melanoma transplant mouse model [56]. Notably, the authors also reported favorable changes of the TME, including an increase in intratumoral CTL and higher levels of proinflammatory cytokines. Similar effects were observed during early BRAFi treatment for metastatic melanoma patients, indicative of the favorable properties of triple combination therapy on the TME [44]. In line, subsequent reports observed superior anti-tumor effects of a triple combination therapy comprising the BRAFi dabrafenib, the MEKi trametinib and a PD-1 blocking mAb as compared to either monotherapy in a murine autochthonous BRAF^V600E^ melanoma model [32, 57]. These studies also showed that triple combination therapy was associated with increased tumor antigen and MHC-I expression and concomitantly a stronger influx of TIL and increased cellular anti-tumor activity as reflected by enhanced IFN-γ expression [32]. Further studies revealed that BRAFi may not only synergize with CPI, but also enhanced anti-tumor activity of adoptive T cell transfer [58] and antibody-mediated CCL2 neutralization to counteract tumor infiltration by immunomodulatory cell types [59]. The synergistic effects of MAPKi with anti-PD-1/PD-L1 mAb were not limited to preclinical BRAF-mutated melanoma models, but could also be observed in BRAF wild-type tumor models, providing evidence that MEKi modulate T cell function via paradoxical ERK activation [34, 60].

These studies were complemented by Deken and coworkers who observed that the synergistic effects of a triple combination therapy of BRAF/MEKi plus anti-PD1 inhibitors were dependent on the CTL recruitment in a BRAF^V600E^/PTEN^−/−^-driven melanoma mouse model [31]. Similar to previous observations from human biopsies, the authors found that short-term BRAF/MEK inhibition enhanced tumor immune cell infiltration but decreased over time [31]. Importantly, and in contrast to the works of Hu-Lieskovan, Deken et al*.* did not apply continuous and concomitant MAPKi therapy but discontinued BRAF/MEKi therapy after a 2-week run-in phase on the premise to avoid the negative immunomodulatory effects of long-term BRAF/MEKi therapy, thus providing insights into optimal timing of co-treatment. In a more recent study, the favorable effects of combining MAPKi and CPI were again confirmed, albeit the authors observed even more profound tumor control using a quadruple combination regimen with DT and combined CPI therapy in a BRAF^V600E^-mutant murine melanoma model [61]. By contrast, combined application of anti-CTLA-4 mAb with the BRAFi vemurafenib in an autochthonous BRAF^V600^/PTEN^−/−^ melanoma model yielded a decrease in TIL questioning the synergistic effects of BRAFi and anti-CTLA4 mAb [62].

In contrast to concomitant multi-drug combination regimens for tumor therapy, sequencing strategies to overcome innate and acquired therapy resistance have been investigated less extensively in preclinical murine studies. These sequential regimens have been proposed to prevent the development of cross-resistance by combining complementary mechanisms of action and allowing one therapy to prime the responsiveness to the other, while reducing the risk of intolerable toxicities. In this regard, it has been shown that a lead-in of anti-PD-1/PD-L1 mAb treatment before MAPKi application improved anti-tumor immunity and survival as compared to single-agent anti-PD-L1 treatment [63]. This observation confirms the long-term favorable effects of CPI that may outweigh the short-term effects of MAPKi in the setting of advanced metastatic disease [51, 64]. Importantly, the authors were also able to show that the sequential application of CPI and MAPKi favored durable (intracranial) tumor responses [63]. As intracranial response rates of MAPKi are generally found to be weaker than extracranial responses, secondary acquired resistance emerges preferentially in the brain, which underscores the importance of this treatment strategy in providing durable intracranial tumor control. Last, preclinical studies suggest that tumor resistance towards MAPKi emerges as a result of the continuous administration of BRAF/MEK inhibitors [65] and may be reverted by short-term drug cessation or intermittent dosing regimens of MAPKi [65, 66].

## Safety and efficacy of triple combination regimens with CPI and MAPKi in clinical trials

Considering the complementary response kinetics of BRAF/MEKi and CPI in clinical settings, as well as the pre-clinical evidence for the synergistic effects of both treatment modalities, it was hypothesized that the optimal combination or sequential application of CPI and MAPKi may provide additional clinical benefit as evaluated in a number of clinical trials (reviewed in [18, 67–69]. Moreover, it has been proposed that the combined application of MAPKi and CPI may help to maintain tumor responses and to overcome MAPK-resistance that is driven both by the strong transcriptional heterogeneity of melanoma and immune-mediated mechanisms of acquired resistance, such as the upregulation of PD-L1 during MAPKi therapy [70]. In the following we will summarize the evidence on the efficacy of both concurrent (see Table 1A, B) and sequential applications (see Table 1C, D) of MAPKi and CPI that has been gathered so-far in clinical trials.

**Table 1:** Selection of clinical trials investigating the safety and efficacy of a combined MAPK-directed and CPI therapy

National Clinical Trial (NCT) Number	Title	Regimen	No. of patients	Median follow-up (months)	Main outcomes	AE	Start Date
A Concomitant administration of MAPKi-directed and CPI therapy for BRAF^V600^-mutant melanoma							
NCT 01400451 [71]	Phase I trial of vemurafenib (VEM) and IPI in subjects with metastatic melanoma	IPI 3 mg/kg + VEM 960 mg vs. IPI 3 mg/kg + VEM 720 mg vs. VEM 720 mg	12	Not given	Trial was terminated due to unexpected grade 2/3 hepatotoxicity	7/10 (70%) grade 2–3 hepatotoxicity	11/2011
NCT 01767454 [73, 118]	Phase I study of dabrafenib (DAB) and trametinib (TRA) in combination with IPI for unresectable or metastatic melanoma	IPI 3 mg/kg + DAB 150 mg vs. DAB 100 mg + TRA 1 mg followed by IPI 3 mg/kg	16	Not given	Trial was closed following 2 cases of dose-limiting toxicity (DLT) in the triple therapy arm (colitis with subsequent perforation); no efficacy data reported	No DLT in the double therapy arm; 28.5% DLT observed in the triple therapy arm	02/2013
NCT 01656642 [80, 99, 100]	Phase Ib study of Atezolizumab (ATE) in combination with VEM or VEM plus cobimetinib (COB) in participants with metastatic melanoma	VEM 960 mg + COB 60 mg (28d), followed by VEM 720 mg + COB 60 mg + ATE 840 mg vs. VEM 960 mg + COB 60 mg + Placebo; cohorts 1–3 tested for different run-in periods of VEM/COB	56	29.9	ORR: 71.8% with CR in 20.5%; median DOR: 17.4 mo; median PFS: 12.9 mo; median OS: not reached	Grade 3/4 treatment-related AE: 66.7%; AE leading to discontinuation in 28.2%; three fatal events were reported	08/2012
NCT 02908672 (IMspire150) [75]	Phase III, double-blinded, randomized, placebo-controlled study of ATE + COB + VEM vs. placebo + COB + VEM in previously untreated patients with unresectable locally advanced or metastatic melanoma (IMspire150)	VEM 960 mg + COB 60 mg (21d), followed by VEM 720 mg + COB 60 mg + ATE 840 mg vs. VEM 960 mg + COB 60 mg (21d) + Placebo	514	18.9	ORR: 66.3% vs. 65.0% Median DOR: 21.6 vs. 12.6 mo Median PFS: 15.1 vs. 10.6 months (HR: 0.78, p = 0.025) Median OS: not reached in interim analysis	Grade 3/4 treatment-related AE: 79% vs. 73%; AE leading to discontinuation of all treatments: 13% vs. 16%	01/2017
NCT 02130466 (MK-3475–022/KEYNOTE-022) [81]	Phase I Study to assess the safety and efficacy of MK-3475 in combination with TRA and DAB in subjects with advanced melanoma	Pembrolizumab (PEM) 2 mg/kg + DAB 150 mg + TRA 2 mg	15	27.0	ORR: 73%; ongoing response: 40%; median PFS: 15.4 months	Grade 3/4 treatment-related AE: 73%; AE resulting in discontinuation: 33%; DLT were reported in 20% (neutropenia and impaired liver functions); no treatment related deaths	05/2014
NCT 02130466 (KEYNOTE-022) [81, 82]	A Phase II study to assess the safety and efficacy of MK-3475 in combination with TRA and DAB in subjects with advanced melanoma	PEM 2 mg/kg + DAB 150 mg + TRA 2 mg vs. placebo + DAB 150 mg + TRA 2 mg	120	9.6	ORR: 63.3% vs. 71.7%; ongoing response: 44.7%; median DOR: 18.7 vs. 12.5 mo; median PFS: 16.0 vs. 10.3 mo (HR: 0.66, p = 0.043, ns); median OS: not reached	Treatment-related AE of grade 3 or higher: 58.3% vs. 26.7%; discontinuation of treatment: 25 vs. 15%; one treatment-related death due to pneumonitis	05/2014
NCT 02130466 (Extended follow-up) [76]	A phase II study to assess the safety and efficacy of MK-3475 in combination with TRA and DAB in subjects with advanced melanoma	PEM 2 mg/kg + DAB 150 mg + TRA 2 mg vs. placebo + DAB 150 mg + TRA 2 mg	120	36.6	ORR: 63% vs. 72%; ongoing response: 29% vs. 9%; median DOR: 25.1 vs. 12.1 mo; median PFS: 16.9 vs. 10.7 mo (HR: 0.53); median OS: NR vs. 26.3 mo	Treatment-related AE of grade 3 or higher: 58% vs. 25%	05/2014
NCT 02967692 (COMBI-i) [83]	Part 1 and 2 of the randomized, double-blind, placebo-controlled phase III study comparing the combination of PDR001, DAB and TRA vs. placebo, DAB and TRA in previously untreated patients with unresectable or metastatic melanoma	Spartalizumab (SPA) 400 mg + DAB 150 mg + TRA 2 mg vs. placebo + DAB 150 mg + TRA 2 mg	36	24.3	ORR: 78%, with 44% showing CR; median PFS: 23 mo; median OS: not reached	Grade 3/4 treatment-related AE: 72%; AE leading to permanent discontinuation: 17%; no treatment-related deaths; DLT were reported in 11% during initial safety run-in	02/2017
NCT 02967692 (COMBI-i) [84, 85]	Part 3 of the randomized, double-blind, placebo-controlled phase III study comparing the combination of PDR001, DAB and TRA vs. placebo, DAB and TRA in previously untreated patients with unresectable or metastatic melanoma	SPA 400 mg + DAB 150 mg + TRA 2 mg vs. placebo + DAB 150 mg + TRA 2 mg	532	27.2	ORR: 69% vs. 64%; median DOR: NR vs. 20.7 mo; median PFS: 16.2 vs. 12.0 mo (HR: 0.82, p = 0.042)	Treatment-related AE of grade 3 or higher: 55% vs. 33%; permanent discontinuation of treatment: 12 vs. 8%	02/2017
NCT 03625141 (TRICOTEL) [95]	A study evaluating the safety and efficacy of COB plus ATE in BRAF wild-type melanoma With Central Nervous System Metastases and COB plus ATE and VEM in BRAFV600-Mutated melanoma with central nervous system (CNS) metastases	COB 60 mg + ATE 840 mg (BRAF wild-type) vs. VEM 960 mg + COB 60 mg (28d run-in) + ATE 840 mg (BRAF^V600^ mutant)	15 + 65	6.2 vs. 9.7	Intracranial ORR: 27% vs. 42%	Treatment-related AE of grade 3 or higher: 53% vs. 68%; one treatment-related death in triple combination group	08/2018
NCT 02902042 (IMMU-TARGET) [129]	Randomized phase I/II open label, multicenter study of encorafenib (ENC) plus binimetinib (BIN) and PEM in patients with unresectable or metastatic Melanoma (IMMU-TARGET)	ENC 450 mg + BIN 45 mg + PEM 200 mg vs. PEM 200 mg only	15	25	ORR: 64%; 12-month PFS: 41%	Treatment-related AE of grade 3 or higher: 53%; DLT were not observed	04/2018
NCT 02910700 (TRIDENT) [130]	A Phase II study of the TRIplet combination of DAB, nivolumab (NIVO), and TRA (TRIDeNT) or BIN, ENC and NIVO (TRIBECA) in patients with metastatic melanoma	NIVO 3 mg/kg + DAB 150 mg + TRA 2 mg (A) vs. NIVO 3 mg/kg + TRA 2 mg (B) vs. NIVO 3 mg/kg + ENC + BIN (C)	27	18.4	Preliminary results: ORR: 92%; ORR in NIVO-refractory patients: 88%; median PFS: 8.5 mo vs. 8.2 mo for NIVO-refractory patients	Preliminary results: 78% of patients showed treatment-related AE of grade 3 or higher; discontinuation due to toxicity in 22%	12/2016
B Concomitant administration of MAPK-directed and CPI therapy for BRAF wild-type melanoma							
NCT 02027961 [79]	Phase I open-label study of safety and tolerability of durvulumab (DUR) in combination with DAB and TRA or with TRA alone in subjects with metastatic or unresectable melanoma	DUR 10 mg/kg + DAB 150 mg + TRA 2 mg vs. DUR 10 mg/kg + TRA 2 mg (concurrent) vs. TRA 2 mg followed by DUR 10 mg/kg (sequential)	68	20.8	ORR: 69.2% vs. 20.0% vs. 31.8% Ongoing response: 50% vs. 75% vs. 42.9%; median DOR: 15.5 vs. NA vs. 8.7 mo; median PFS: 11.2 vs. 4.9 vs. 5.9 mo; median OS: 31.4 vs. NA vs. 21.7 mo	Grade 3/4 treatment-related AEs: 69% vs. 80% vs. 73%; AE leading to discontinuation: 46.2% vs. 35% vs. 50%; no deaths related to toxicity	12/2013
NCT 03273153 [86]	Phase III, open-label, multicenter, two arm, randomized study to investigate the efficacy and safety of COB plus ATE versus PEM in patients with previously untreated advanced BRAF wild-type melanoma	COB 60 mg + ATE 840 mg vs. PEM 200 mg	446	NA	ORR: 26 vs. 32%; median PFS: 5.5 vs. 5.7 mo; median OS: NA vs. 29.3 mo	Grade 3/4 treatment-realted AE: 67% vs. 33%; AE leading to discontinuation of all treatments: 12% vs. 6%	12/2017
C Optimal treatment sequencing upon disease progression for BRAF/MEK-directed targeted therapy and CPI therapy							
NCT 02224781 (DREAMSeq) [124, 125]	Phase III trial (DREAMseq) (Doublet, randomized evaluation in advanced melanoma sequencing)	IPI 3 mg/kg + NIVO 1 mg/kg followed by DAB 150 mg + TRA 2 mg (Arm A) vs. DAB 150 mg + TRA 2 mg followed by IPI 3 mg/kg + NIVO 1 mg/kg (Arm B)	265	27.7	ORR: 46% vs. 43% (Step 1) and 47.8% vs. 29.6% (Step 2); median duration of response: NR vs. 12.7 mo; median PFS: 11.8 vs. 8.5 mo; 2-year PFS rate: 41.9% vs. 19.2%; 2-year OS rate: 71.8% vs. 51.5%;	Treatment-related AE grade 3 or higher: 59.5% vs. 53.8%. Three treatment related deaths (two in Arm A, one in Arm B)	08/2014
NCT 02631447 (SECOMBIT) [126]	Three arms prospective, randomized phase II study to evaluate the best sequential approach With Combo Immunotherapy (IPI/NIVO) and Combo Target Therapy (ENC + BIN) in patients With Metastatic Melanoma and BRAF Mutation (SECOMBIT)	ENC 450 mg + BIN 45 mg until PD, then NIVO 1 mg/kg + IPI 3 mg/kg (Arm A) vs. NIVO + IPI for 4 doses, followed by NIVO 3 mg/kg, until progressive disease (PD) then ENC + BIN (Arm B) vs. ENC + BIN for 8 weeks followed by NIVO + IPI for 4 doses, then NIVO until PD; then ENC + BIN (Arm C)	209	32.2	ORR: 87% vs. 44.9% vs. 82.4% (Step 1); 25.7% vs. 57.9% vs. 62.2% (Step 2); 2-year OS: 65% vs. 73% vs. 69%; 2-year PFS rate: 46% vs. 65% vs. 57%	trAE grade 3 or higher: 39% vs. 59% vs. 38%. AE leading to discontinuation occurred in 10% vs. 10% vs. 9%; no fatal events were being reported	11/2016
D Sequential administration of BRAF/MEK-directed targeted therapy and CPI therapy							
NCT 01673854 [72]	Single arm open-label phase II study of VEM followed by IPI in subjects with previously untreated advanced melanoma	VEM 960 mg administered for 6 weeks followed by IPI 10 mg/kg	46	15.3	no severe hepatotoxicity, but other grade 3/4 AE; median OS: 18.5 mo; median PFS: 4.5 mo	Grade 3/4 AE of the skin (32.6%), GI-tract (21.7%) and hepato-biliary system (4.3%)	09/2012
NCT 02902029 (ImmunoCobiVem) [128]	Phase II, open-label, randomized-controlled trial evaluating the efficacy and safety of sequential of VEM plus COB followed by immunotherapy with ATE for the treatment of patients with unresectable or metastatic BRAF^V600^-mutant melanoma (ImmunoCobiVem)	3 month run-in period with VEM 960 mg + COB 60 mg, followed by VEM 960 mg + COB 60 mg until PD, then ATE 1200 mg vs. 3 months run-in period with VEM 960 mg + COB 60 mg, followed by ATE 1200 mg until PD, then cross-back to VEM 960 mg + COB 60 mg	185	19.0	Arm A: 52% discontinued treatment due to PD and switched to ATE; Arm B: 71% discontinued ATE due to PD and switched to VEM/COB; median PFS was longer in Arm A vs. Arm B (HR: 0.55, p = 0.001); median OS: similar between both arms (HR: 1.22, p = .37)	trAE grade 3 or higher were reported in 55% vs. 64%	11/2016

***Abbreviations***: ATE = atezolizumab; ALT = alanine-aminotransferase; AST = aspartate aminotransferase; BIN = binimetinib; COB = cobimetinib; CR = complete response; DAB = dabrafenib; DOR = duration of response; DLT = dose-limiting toxicity; DUR = durvalumab; ENC = encorafenib;; HR = hazard ratio; IPI = ipilimumab; NA = not available; NIVO = nivolumab; NR = not reached; ns = not significant; ORR = overall response rate; OS = overall survival; PEM = pembrolizumab; PFS = progression-free survival; (tr)AE = (treatment-related) adverse events; TRA = trametinib; VEM = vemurafenib

### Combined application

The first phase I clinical trial that studied the combined application of a CPI (IPI) plus single-agent MAPKi (vemurafenib) for patients with BRAF-mutated metastatic malignant melanoma was terminated due to drastic side effects such as liver toxicity and skin rashes (NCT01400451) [71]. As single-agent BRAFi are known for an adverse toxicity profile and lower response rates as compared to combination MAPKi subsequent studies focused on the additional application of BRAF plus MEKi rather than single-agent BRAFi. Also, toxicity appears to be worse in case of treatment with vemurafenib than dabrafenib, suggesting that the occurrence of specific drug-related AE should be considered in the design of combination therapies [72]. In line, another phase I study (NCT01767454) showed that the combination of DT plus IPI had a better safety profile with regard to hepatotoxicity [73], but resulted in severe cases of immune-associated colitis being most commonly associated with IPI treatment [74].

Due to the adverse toxicity profile reported for combinations with IPI, subsequent trials largely focused on combinations of MAPKi with PD-1/PD-L1 inhibitors. These have shown better manageable toxicity albeit tolerability still remained a critical consideration for triple combination regimens [75–79].

Sullivan and coworkers observed in a phase Ib study including 56 patients with advanced BRAF-mutant melanoma similar response rates (71.8% vs. 76.5%) for the combination of the PD-L1 inhibitor atezolizumab plus the BRAFi/MEKi combination vemurafenib and cobimetinib as compared to vemurafenib and cobimetinib alone, but also observed more durable responses (17.4 vs. 10.6 months) and a longer median PFS (12.9 vs. 10.9 months) with the triple combination [80]. More importantly, biomarker analysis from phase Ib of this trial suggested that the 28-day run-in period of vemurafenib and cobimetinib before initiating additional atezolizumab treatment induced favorable TME changes including enhanced infiltration by CD8^+^ and CD4^+^ T cells [80]. The subsequent IMspire 150 phase III trial (NCT02908672) including 514 patients with unresectable stage IIIC/IV BRAF-mutant melanoma confirmed a significantly prolonged PFS in the triple combination arm (15.1 vs. 10.6 months, hazard ratio, HR: 0.78, p = 0.025) at a median follow-up of 18.9 months due to a longer duration of response (21.0 vs. 12.6 months) and delayed CNS metastasis [75]. Therefore, the combination of atezolizumab, vemurafenib and cobimetinib has been approved for treatment of metastatic melanoma by the FDA. Meanwhile, the median OS (28.8 vs. 25.1 months) and the overall response rate (ORR) (66.3 vs. 65.0%) were similar between both groups, although separation of OS curves at 2 years suggests an emerging benefit over time. Importantly, the triple combination therapy was well tolerated (discontinuation: 12.6 vs. 15.7%). However, somewhat more serious treatment-related adverse events (trAE) have been reported in the triple combination therapy arm (79% vs. 73%), including elevated creatine phosphokinase, diarrhea, rash, arthralgia, pyrexia and elevated liver enzymes [75]. While it remains unclear to which extent the initial vemurafenib plus cobimetinib run-in period contributed to the efficacy of this triple combination approach, it has been argued that better tolerability might mainly be inferred from the addition of the PD-L1 inhibitor atezolizumab, albeit the lower vemurafenib dose in the CPI arm (720 mg vs. 960 mg in the comparator arm) complicated a direct comparison.

The phase I/II KEYNOTE 022 trial (NCT02130466) investigated the combination of the PD-1 inhibitor pembrolizumab plus DT versus DT only in a cohort of 120 advanced BRAF-mutant melanoma patients [81, 82]. While the primary analysis showed an improved median PFS for the triple therapy cohort (16.0 vs. 10.3 months; HR: 0.66, p = 0.043) the trial did however not reach the pre-specified significance criteria after a median follow-up of 9.6 months. Furthermore, the ORR of the triple therapy group was weaker as compared to DT (63% vs. 72%) which was attributed to adverse prognostic features among patients in this treatment group. However, the addition of pembrolizumab enhanced the rate of complete response (18% vs. 13%) and the duration of response (18.7 vs. 12.5 months) that was observed across all subgroups and particularly in patients with adverse prognostic factors [82]. In addition, analyses from dose-escalation cohorts (n = 15) of the KEYNOTE-022 trial indicated that CD8^+^ T cell infiltration and overall PD-L1 expression from pre-treatment biopsies might serve as valuable predictive factors for response to triple combination regimens [81]. Serious trAE of grade 3 or higher were more frequent among patients in the triple combination cohort (70% vs. 45%) and included pyrexia, elevated liver enzymes, skin rash and pneumonitis. While most AE were manageable with dose modifications, 25% of patients in that group had to discontinue treatment due to serious AE as compared to 15% in the active comparator arm. Notably, a subsequent analysis with extended follow-up (median follow-up of 36.6 months) reported greater improvements in PFS, duration of response (DOR) and OS, which suggests that the benefits of triplet therapy might be more pronounced during additional follow-up (see Table 1) [76].

These data were complemented by the randomized, double-blind, placebo-controlled (RCT) phase III COMBI-I trial (NCT02967692) that evaluated the combination of the investigational anti-PD1 mAb spartalizumab plus the BRAF/MEKi DT [83]. This trial was launched after a successful initial safety-run in with 36 patients that demonstrated high efficacy with an ORR of 78%, 44% of patients showing CR and a median PFS of 23 months. Notably, additional biomarker analyses were able to replicate the preclinical observation in murine models that the favorable immunomodulatory effects of MAPKi were gradually reversed from baseline to 2–3 weeks during treatment and were lost upon tumor progression [83]. Also, the authors were able to show that a higher tumor mutational burden and T cell-inflamed gene expression profiles were associated with a longer PFS. The recently updated data from part 3 of COMBI-I with a median follow-up of 27.2 months largely confirmed these preliminary results in a cohort of 532 patients with advanced BRAF-mutant melanoma [84]. In accordance with the previous KEYNOTE-022 trial, the median PFS (16.2 vs. 12.0 months, HR: 0.82, p = 0.042) and duration of response (not reached, NR vs. 20.7 months) were improved for patients given triple combination regimens. However, the study did not meet its primary endpoint and thus the broad first-line use of this triple combination therapy was not supported by the presented trial results [85]. Also, the ORR between the triple therapy arm and the comparator arm was comparable (69% vs. 64%), while the triple combination group experienced more trAE of grade 3 or higher (55% vs. 33%) and accordingly treatment had to be discontinued more often as compared to the comparator arm (12% vs. 8%) [85].

Altogether the phase III IMspire 150 and COMBI-I trials, as well as the phase II KEYNOTE-022 trial suggested a modest additional benefit of triple combination regimens compared to MAPKi combinations. In particular, IMspire150 was the only trial to show a statistically significant difference in investigator-assessed PFS, which might be inferred from differences in study design (such as the BRAF/MEKi run-in phase for IMspire 150), patient populations and dosing schedules [76, 85, 86] (see Table 1). Triple combination regimens have also been associated with an adverse toxicity profile, that required the discontinuation of treatment in 12–25% of patients. Most importantly, a significant weakness of these trials is that none included an IPI plus Nivo comparator arm, that is currently considered a standard of care with 5-year PFS rates reaching almost 40% and with a benefit particularly for BRAF-mutant melanoma patients [25]. Whilst the combination of IPI + Nivo also showed high rates of severe trAE, the emerging combination of the lymphocyte activation gene-3 blocking antibody relatlimab plus Nivo armed clinicians with an additional treatment option that showed favorable safety profiles [27]. Therefore, the results of these trials do not support the broad first-line use of triple combination regimens for BRAF^V600^ mutant melanoma patients. However, subgroup analyses from these trials indicate that patients with a high disease burden may derive a greater clinical benefit and further biomarker-driven analyses may help to identify these patient populations more precisely [85]. Also, the immunomodulatory properties of MAPKi to enhance the efficacy of immunotherapies other than CPI, such as adoptive cell transfer [87], CAR-T cell therapy and oncolytic viral therapies [88], might present an additional avenue for MAPKi as immunotherapy adjuvants [67, 87–89]. In this regard, inhibition of the immunosuppressive properties of the TME with short-term administration of vemurafenib has been suggested to be a safe and feasible option prior to adoptive transfers of autologous TIL as shown by Borch and coworkers [90].

#### Triple therapy regimen in patients with melanoma brain metastases

Although clinical trials evaluating triple combinations in CPI-naïve patients have yielded mixed results, further analysis indicated that such combinations may result in a substantial benefit for patients with poor prognostic features. In this regard, patients with melanoma brain metastases (MBM) and patients refractory to initial CPI therapy are of particular interest given that standard treatments showed substantially weaker response rates in second-line settings and for patients with MBM [17, 91]. Also, clinically acquired treatment resistance to MAPKi emerges preferentially in the brain, and often precedes extracranial disease progression [92–94]. As exploratory analysis from the IMspire 150 trial demonstrated that the combined application of atezolizumab plus MAPKi delayed the development of MBM and reduced the overall incidence of MBM, this triple combination regimen is currently under further investigation in clinical trials. The open-label, single-arm phase II TRICOTEL trial (NCT03625141) evaluated the efficacy of atezolizumab plus vemurafenib and cobimetinib versus atezolizumab plus cobimetinib in a cohort of 80 patients with either BRAF wild-type (n = 15) or BRAF^V600^-mutant melanoma (n = 65) and both symptomatic or asymptomatic MBM [95]. After a median follow-up of 9.7 months, median intracranial duration of response was 7.4 months with an intracranial ORR of 51% and a median intracranial PFS of 5.8 months in the cohort of patients with BRAF-mutant melanoma, which was similar to patients with asymptomatic MBM and no corticosteroid treatment at baseline. trAE of grade 3 or higher were reported in 68% of patients and 27% had to discontinue any study treatment due to AE. By contrast, intracranial response (27%) and PFS (2.2 months) was substantially shorter in accordingly treated BRAF wild-type patients.

While indirect comparisons with previous trials should be interpreted with caution, given the differences in study design and patient populations, as well as various definitions of symptomatic patients, the results from the TRICOTEL trial suggest that triple combination regimens provide similar rates of intracranial response (58 vs. 59%), while allowing for more durable responses as compared to combined BRAFi and MEKi therapy alone [17]. Also, results of the TRICOTEL trial provide evidence that lead-in with vemurafenib plus cobimetinib may reduce the necessity of corticosteroid use, thereby enhancing efficacy of subsequent CPI therapy. In addition, preliminary results from the ongoing phase II TriDent trial (NCT02910700), that included 17 patients with PD1-refractory MBM, indicated that the triple combination of Nivo and DT conferred promising clinical activity in CPI-refractory patients, while toxicity profiles were consistent with previously reported triple combinations [96]. In particular, the authors observed ORR of 88% in CPI-refractory patients and intracranial response rates of 57% of patients with MBM. The median PFS after a follow-up time of 18.4 months was 8.5 months [96]. In order to address the caveat that none of the trials included IPI plus Nivo as a comparator arm, a recently started phase II clinical trial aims to evaluate triple administration of Nivo, encorafenib and binimetinib versus IPI and Nivo in patients with MBM (NCT04511013).

#### Intermittent administration of MAPKi in triplet combination regimens

In an attempt to reduce the high frequency of trAE seen for continuous combination treatment with MAPKi and CPI, more recent studies investigated the safety and efficacy of short-time, intermittent MAPKi plus CPI regimen. Intermittent MAPKi application was previously found to act synergistically with anti-PD1 mAb in pre-clinical models and delayed the induction of secondary acquired resistance [65, 66]. In contrast to this hypothesis, the open-label phase II S1320 trial (NCT02196181), which evaluated intermittent versus continuous application of DT in patients with advanced BRAF^V600^-mutant melanoma, showed that intermittent dosing neither improved PFS (median PFS: 5.5 months vs. 9.0 months, p = 0.064) nor reduced AE [97]. The phase IIb IMPemBra trial (NCT02625337) evaluated the combination of pembrolizumab with intermittent DT versus the continuous application of both MAPKi agents in 32 patients with advanced BRAF^V600^-mutant melanoma [77, 98]. As opposed to the disappointing results from the previous S1320 trial, preliminary data from this trial suggest that the short-term addition of intermittent DT to pembrolizumab is more feasible, tolerable and effective as the continuous triple combination and thus final results from this trial are eagerly awaited [77, 98].

#### Combined application of MAPKi and CPI for patients with BRAF wild-type melanoma

Patients with wild-type BRAF melanoma do not respond to BRAFi, but pre-clinical studies indicate that these may profit from MEKi therapy [34, 60]. These observations from BRAF wild-type tumor models did however not translate into clinical practice so-far [99, 100]. In particular, co-application of the anti-PD-L1 mAb atezolizumab and the MEKi cobimetinib failed to improve PFS as compared to the anti-PD-1 mAb pembrolizumab monotherapy (5.5 vs. 5.7 months; HR: 1.15) in a phase 3 clinical trial of 466 patients with untreated BRAF wild-type advanced melanoma (NCT03273153), and significantly more patients developed serious AE (44.1 vs. 20.8%) [86]. These results are in line with a previous phase I trial that evaluated the combination of the anti-PD-L1 mAb durvalumab in combination with DT in a cohort of 26 patients with BRAF^V600^-mutant melanoma and 42 patients with BRAF wild-type melanoma (NCT02027961) [79]. While the authors reported an ORR of 69.2% with almost 90% of sustained responses and a median duration of response of 15.5 months for patients with BRAF^V600^-mutant melanoma, results from the BRAF wild-type melanoma cohort suggested that the combined application of durvalumab and trametinib did not improve response rates as reflected by a short median PFS of only 5.9 months [79]. Limited antitumor activity for this combination therapy in BRAF wild-type melanoma patients was also confirmed in part 4 and 5 of the KEYNOTE-022 trial (NCT02130466) that investigated both concurrent and intermittent administration of trametinib plus pembrolizumab. Both treatments yielded similar safety results, but patients in the intermittent regimen group showed a higher ORR (30% vs. 20%) [101].

#### Triple combination regimens in the neoadjuvant setting for resectable BRAF-mutant melanoma

Triple combination therapy has not only been investigated in the metastatic, but also in the neoadjuvant setting. In this setting, CPI or MAPKi are administered prior to definitive surgery. Neoadjuvant treatment regimens for resectable advanced stage III and stage IV melanoma are increasingly coming into focus of clinical investigations given the promising results of the recent OpACIN [102] and OpACIN-neo [103, 104] trials that are backed by preclinical data demonstrating that the presence of a clinical detectable tumor burden improves antigen priming and enhances subsequent anti-tumor immune responses [105–107]. In particular, the OpACIN trial investigated the efficacy of IPI 3 mg/kg plus Nivo 1 mg/kg given either adjuvant as four courses after surgery or split into two courses prior to and after surgery for resectable stage III melanoma patients [102]. After more than 5 years of follow-up RFS and OS rates for neoadjuvant IPI plus Nivo were 70% versus 60% and 90% versus 70%, highlighting the durability of responses upon neoadjuvant IPI + Nivo [108]. The subsequent multicenter, randomized phase II OpACIN-neo trial complemented these data investigating neoadjuvant IPI plus Nivo in three dosing schedules [104]. Here, the administration of IPI 1 mg/kg and Nivo 3 mg/kg led to pathological response rates (pRR) of 77%, suggesting similar efficacy data but lower rates of grade 3–4 toxicity (20% vs. 40%) as compared to IPI 3 mg/kg and Nivo 1 mg/kg. In addition to the substantial prolongation of RFS and OS, both OpACIN trials reported that pathological response was a strong surrogate for tumor recurrence, with only 3/64 patients showing a tumor relapse after initial pathological response as opposed to 14/21 without pathological response [108].

Results from recently presented trials in patients with resectable stage III or oligometastatic stage IV melanoma confirmed these promising results for neoadjuvant Nivo plus relatlimab [109]. The neoadjuvant application of DT was investigated in the phase II NeoCombi trial for patients with resectable BRAF-mutant stage III melanoma. In line with neoadjuvant CPI therapy, pRR was high (100%) and 49% achieved pCR [110]. As opposed neoadjuvant CPI therapy, rates of tumor relapse for patients with pathological response were considerably higher (8/17), and RFS weaker as compared to previously reported data for adjuvant DT.

Despite the strong efficacy data for neoadjuvant treatment regimens, there remains a need to enhance treatment response for those patients that did not initially show pathological response. Due to the complementary effects of MAPKi and CPI and the high pRR among patients given neoadjuvant DT it has been reasoned that neoadjuvant triple combination therapies might enhance treatment responses in these patients. However, preliminary results have yet only been reported for the NeoTrio trial (NCT02858921), a randomized trial that compared the neoadjuvant single-agent pembrolizumab with the sequential application of pembrolizumab with DT or the concurrent application of DT plus pembrolizumab in patients with resectable BRAF-mutant stage III melanoma. In particular it has been reported that pRR and pCR were highest in the triple combination arm as compared to the sequential arm or the single-agent arm [111]. However, and in line with previous trials in the non-resectable, metastatic setting, patients given triplet combination therapies more often presented with severe trAE that led to treatment interruption or discontinuation. Similar to previous trials on optimal treatment sequencing in the metastatic setting, short-course of DT prior to anti-PD1 therapy did not improve pRR in the NeoTrio trial. Given the short follow-up time and the lack of additional data on triple combination therapies in the neoadjuvant setting (the single-arm phase II Neo-VC trial which aimed to investigate the neoadjuvant application of vemurafenib and cobimetinib plus atezolizumab was closed early due to poor recruitment) and the limited number of patients included in the NeoTrio trial (n = 60) further follow-up data, as well as results from other ongoing neoadjuvant trials (see Table 2D) will be required to assess the efficacy of triplet combination therapies as compared to currently applied MAPKi and CPI regimens.

**Table 2:** Ongoing clinical trials investigating the efficacy of a combination of MAPK-directed targeted therapy and CPI therapy

National Clinical Trial (NCT) Number	Title	Status	Conditions	Interventions	Clinical Phase	Start Date
A BRAF-mutant melanoma: concomitant administration of MAPKdirected and CPI therapy						
NCT 04657991	Randomized, double-blind study of ENC and BIN plus PEM versus placebo plus PEM	Recruiting	Unresectable or metastatic BRAF^V600^-mutant melanoma	ENC 450 mg + BIN 45 mg + PEM 200 mg vs. PEM 200 mg only	3	12/2020
NCT 04511013 (SWOG S2000)	Randomized trial of ENC plus BIN plus NIVO vs. IPI plus NIVO in BRAF^V600^-mutant melanoma with MBM	Recruiting	Metastatic cutaneous melanoma with MBM	ENC 450 mg + BIN 45 mg + NIVO 3 mg/kg vs. IPI 3 mg/kg + NIVO 1 mg/kg	2	12/2020
NCT 04657991	Randomized, double-blind study of ENC and BIN plus PEM versus placebo plus PEM	Recruiting	Metastatic or unresectable locally advanced BRAF^V600E/K^-mutant- melanoma	ENC 450 mg + BIN 45 mg + PEM 200 mg vs. PEM 200 mg only	3	01/2021
B BRAF-mutant melanoma: sequential administration of MAPK-directed and CPI therapy						
NCT 03235245	MAPK-targeted therapy followed by CPI (IPI and NIVO) vs. IPI plus NIVO (EORTC randomized study (EBIN)	Recruiting	Unresectable, BRAF^V600^-mutant stage III melanoma and BRAF^V600^-mutant stage IV melanoma	ENC 450 mg + BIN 45 mg for 12 weeks, followed by IPI 1 mg/kg + NIVO 3 mg/kg vs. IPI 1 mg/kg + NIVO 3 mg/kg only	2	10/2018
NCT 02625337 (IMPemBra)	Comparison of PEM with intermittent/short-term dual MAPK inhibition plus PEM	Recruiting	BRAF^V600^-mutant stage IV melanoma	PEM 200 mg vs. short-term DAB 150 mg + TRA 2 mg + PEM 200 mg vs. intermediate DAB + TRA + PEM vs. long-scheme DAB + TRA + PEM	2	12/2015
NCT 04655157	A Multi-center phase I/II open label study to evaluate safety and efficacy in participants with ENC alone and with BIN in combination with NIVO and low-dose IPI. (QUAD 01: quadruple melanoma therapy)	Recruiting	Unresectable or metastatic BRAF^V600^-mutant melanoma high-risk patients	ENC 300 mg + IPI 1 mg/kg + NIVO 3 mg/kg vs. ENC 450 mg + BIN 45 mg + IPI 1 mg/kg + NIVO 3 mg/kg	1/2	02/2021
C BRAF-wild-type: sequential or concomitant administration of MAPK-directed and CPI therapy						
NCT 03149029	Abbreviated MAPK-targeted therapy plus PEM	Active	Unresectable or metastatic melanoma	PEM + DAB + TRA (BRAF^V600^-mutant) vs. PEM + TRA (BRAF wild-type)	2	11/2017
D Administration of MAPK-directed and CPI therapy in a neoadjuvant setting						
NCT 03554083	Neoadjuvant therapy: A pilot clinical trial	Recruiting	High-risk stage III BRAF^V600^-mutant melanoma	VEM + COB + ATE for up to 3 months followed by surgery and ATE for up to 8 cycles vs. COB + ATE for up to 3 months followed by surgery and ATE for up to 8 cycles	2	06/2018
NCT 02858921	Phase II, randomised, open label study of neoadjuvant DAB, TRA w/o and with PEM (NeoTrio)	Recruiting	BRAF^V600^-mutant resectable stage IIIB/C melanoma	DAB 150 mg + TRA 2 mg (1 we) plus consecutive PEM 2mg/kg for 6 we followed by surgery and PEM 2 mg/kg for 46 we vs. concurrent DAB 150 mg + TRA 2 mg + PEM 200 mg for 6 we followed by surgery and PEM 200mg for 46 we vs. PEM 200 mg only with surgery at week 6	2	11/2017
NCT 04722575	NEOadjuvant Plus Adjuvant Therapy with VEM, COB, and ATE applied in combination or sequentually	Recruiting	High-risk resectable melanoma (stage III B-D and oligometastatic stage IV), both BRAF wild-type and BRAF^V600^-mutant	VEM 960 mg + COB 60 mg followed by surgery and ATE 1200 mg vs. VEM 720 mg + COB 60 mg for 6 we and 2 cycles of ATE 840 mg followed by surgery and ATE 1200 mg vs. COB 60 mg + ATE 840 mg for 6 we followed by surgery and ATE 1200 mg	2	12/2020

**Abbreviations**: ATE = atezolizumab; ALT = alanine-aminotransferase; AST = aspartate aminotransferase; BIN = binimetinib; COB = cobimetinib; CR = complete response; DAB = dabrafenib; DOR = duration of response; DLT = dose-limiting toxicity; DUR = durvalumab; ENC = encorafenib;; HR = hazard ratio; IPI = ipilimumab; NA = not available; NIVO = nivolumab; NR = not reached; ns = not significant; ORR = overall response rate; OS = overall survival; PD = progressive disease; PEM = pembrolizumab; PFS = progression-free survival; (tr)AE = (treatment-related) adverse events; TRA = trametinib; we = week; VEM = vemurafenib

### Sequential administration of MAPKi and CPI for BRAFV600-mutant melanoma

As higher toxicity levels of triple therapies may limit their applicability it has been reasoned that a sequential treatment of CPI and MAPKi may reduce overall toxicity but exert similar efficacy rates. However, initial investigations testing sequential regimens with vemurafenib followed by IPI, provided disappointing results in terms of efficacy with ORR of only 32.6%, although tolerability was substantially improved [72]. In addition, it has previously been shown that response to CPI is considerably weaker after MAPKi failure, presumably due to the emergence of cross-resistance [112, 113]. As it remains unclear when cross-resistance during MAPKi treatment emerges, the sequential administration of MAPKi and CPI faces multiple challenges, including that on the one hand administering CPI first might not take advantage of the immunomodulatory effects of MAPKi on the tumor TME, while on the other hand delayed CPI administration at the time of disease progression following MAPKi might be too late with regard to emerging cross-resistance [69]. In order to account for the differences in tumor biology and subsequent disease outcome upon first-line treatment failure, we will in the following distinguish trials determining optimal treatment sequencing strategies only after disease progression (Table 1C) from trials investigating sequential treatment efficacy in general using the technique of planned switch from MAPKi to CPI therapy (Table 1D).

Optimal treatment sequencing strategies have first been evaluated in retrospective studies demonstrating that responses to second line therapy were typically worse than responses to first-line treatment, regardless of treatment modality [112, 114]. While retrospective investigations initially yielded mixed results [115–118] regarding the question which up-front regimen might be most beneficial for patients with BRAF^V600^ mutant metastatic melanoma, recent evidence rather showed that the up-front use of CPI before MAPKi results in better survival outcomes [118–123].

These favorable outcomes for the upfront use of CPI have also been confirmed by two pivotal randomized-controlled phase II/III trials (see Table 1C). In particular, the randomized-controlled phase III DREAMSeq trial (NCT02224781) compared the upfront use of IPI + Nivo (Arm A) with the combination of DT (Arm B) in 265 treatment-naïve BRAF^V600^-metastatic melanoma patients [124]. At disease progression patients were enrolled in step 2 of the study to receive the alternate therapy, DT (Arm C) or IPI + Nivo (Arm D). After a median follow-up time of 27.7 months data demonstrated a clinically significant benefit for patients given upfront IPI + Nivo with superior 2-year OS rates (71.8% vs. 51.5%) that were observed among all clinical subgroups examined, including those previously suggested to have better outcomes with MAPKi. Most notable, even for patients with highly aggressive disease progression the upfront use of CPI was not found to be inferior to MAPKi. This observation indicated that the therapeutic outcomes might be improved by switching early to second-line MAPKi in patients who appear not to be responding to front-line IPI plus Nivo [124]. Reasons for the superior efficacy of the first CPI sequence were (a) the increased durability of responses (88% vs. < 50%), (b) its ability to delay or prevent the development of MBM, and (c) the comparable efficacy of MAPKi in the second-line setting as compared to second-line CPI therapy. The latter observation is backed by preclinical data showing that CPI may enhance BRAF^V600^-mutated melanoma responsiveness to MAPKi [63]. Meanwhile, toxicity profiles were largely comparable to the previously reported rates with 59% of serious AE in the IPI plus Nivo arm and 53% for DT [125].

In line, the phase II SECOMBIT trial (NCT02631447) confirmed superior 2-year OS rates (73% vs. 65%) for the sequential regimen with upfront IPI plus Nivo as compared to upfront encorafenib plus binimetinib in a cohort of 209 patients with advanced BRAF^V600^-mutant melanoma [126]. However, it is noteworthy that this trial did not formally test for significant differences between each treatment arm. Importantly, the SECOMBIT trial also included a sandwich regimen as a third arm in order to evaluate the clinical relevance of the immunomodulatory properties of MAPKi using a 8-week run-in phase of encorafenib plus binimetinib, a subsequent switch to IPI plus Nivo until disease progression and re-initiation of encorafenib plus binimetinib thereafter. This sandwich approach with BRAF/MEKi lead-in followed by IPI plus Nivo demonstrated clinical benefit and is currently evaluated in the randomized phase II EORTC EBIN trial (NCT03235245) and the phase II COWBOY trial (NCT02968303). Meanwhile, tolerability in all treatment arms of SECOMBIT trial was consistent with previous reports, and more notable the sandwich regimen showed no safety concerns [126].

As compared to the aforementioned trials evaluating optimal treatment sequencing upon disease progression, trials using the technique of planned switch from MAPKi to CPI rather evaluated overall treatment efficacy regardless of disease progression. Here, a single-arm phase II trial comprising 46 patients with advanced melanoma reported that an initial 6 week lead-in phase of vemurafenib before starting IPI 10 mg/kg was associated with a lower rate of severe liver enzyme elevations, but yet promising response rates and median OS of 18.5 months [127]. In addition, the ongoing ImmunoCobiVem trial (NCT02902029) investigates the efficacy of sequential administration of the MAPKi vemurafenib plus cobimetinib with the PD-L1 inhibitor atezolizumab in untreated BRAF^V600^-mutant melanoma patients. In accordance with previous results reported in the SECOMBIT trial, preliminary data indicate that an early switch from vemurafenib and cobimetinib to atezolizumab is feasible and safe, although tumor-control achieved during run-in was maintained only in a subset of patients on subsequent CPI monotherapy [128] (see Table 1D).

Overall, the recent results from prospective phase II/III studies have unequivocally shown that BRAF^V600^-mutant patients with metastatic disease and disease progression after front-line therapy achieve significantly better survival outcomes after front-line IPI plus Nivo therapy and therefore this combination should be favored as first-line therapy whenever feasible. Although cross-study comparisons should be carefully interpreted these data also indicate that the efficacy of upfront IPI plus Nivo, followed by BRAF/MEKi upon relapse is at least as effective as the previously reported triple combination therapies. Further investigations will be necessary to address the question of the most efficient and tolerable treatment regimen particularly for patients with highly aggressive disease. Here, the pending results from prospective trials investigating treatment regimens with short-term MAPKi run-in might give meaningful insights both into the optimal duration of the lead-in phase to avoid the development of cross-resistance and their potential to synergize with subsequent CPI without causing significant toxicity [63]. Owing to the emerging role of neo-adjuvant melanoma therapy and the impressive results reported recently [103], the application of sequential triple therapy is also currently being investigated in a neo-adjuvant setting of high-risk resectable stage III melanoma, which might enable valuable insights into the efficacy of triple therapy in reducing tumor burden prior to surgical removal (see Table 2D).

## Mechanisms of CPI and TT cross-resistance in advanced melanoma

Despite the recent advances in optimizing treatment combinations and sequencing regimens employing combinations of clinically approved MAPKi and CPI, approximately 50% of patients will acquire secondary resistance upon front-line treatment and thus require effective treatment options in subsequent lines of treatment [25]. Secondary acquired resistance is commonly observed in 70–80% of patients given MAPKi, but also emerges in approximately 20–30% of patients treated with CPI. Importantly, recent preclinical evidence has demonstrated that secondary acquired resistance to MAPKi might jeopardize treatment response to subsequent CPI due to the immune-evasive character of the TME [28, 131–134]. This observation suggests that CPI should be administered before patients develop resistance to MAPKi in order to avoid the development of cross-resistance. While it has previously been observed that both MAPKi and IPI plus Nivo also exert anti-tumor activity after previous anti-PD-1 mAb treatment failure [135], response rates in these second line settings remained low as compared to upfront treatments and reflect the observation that secondary resistance will eventually also affect CPI therapy.

**Fig. 2:**
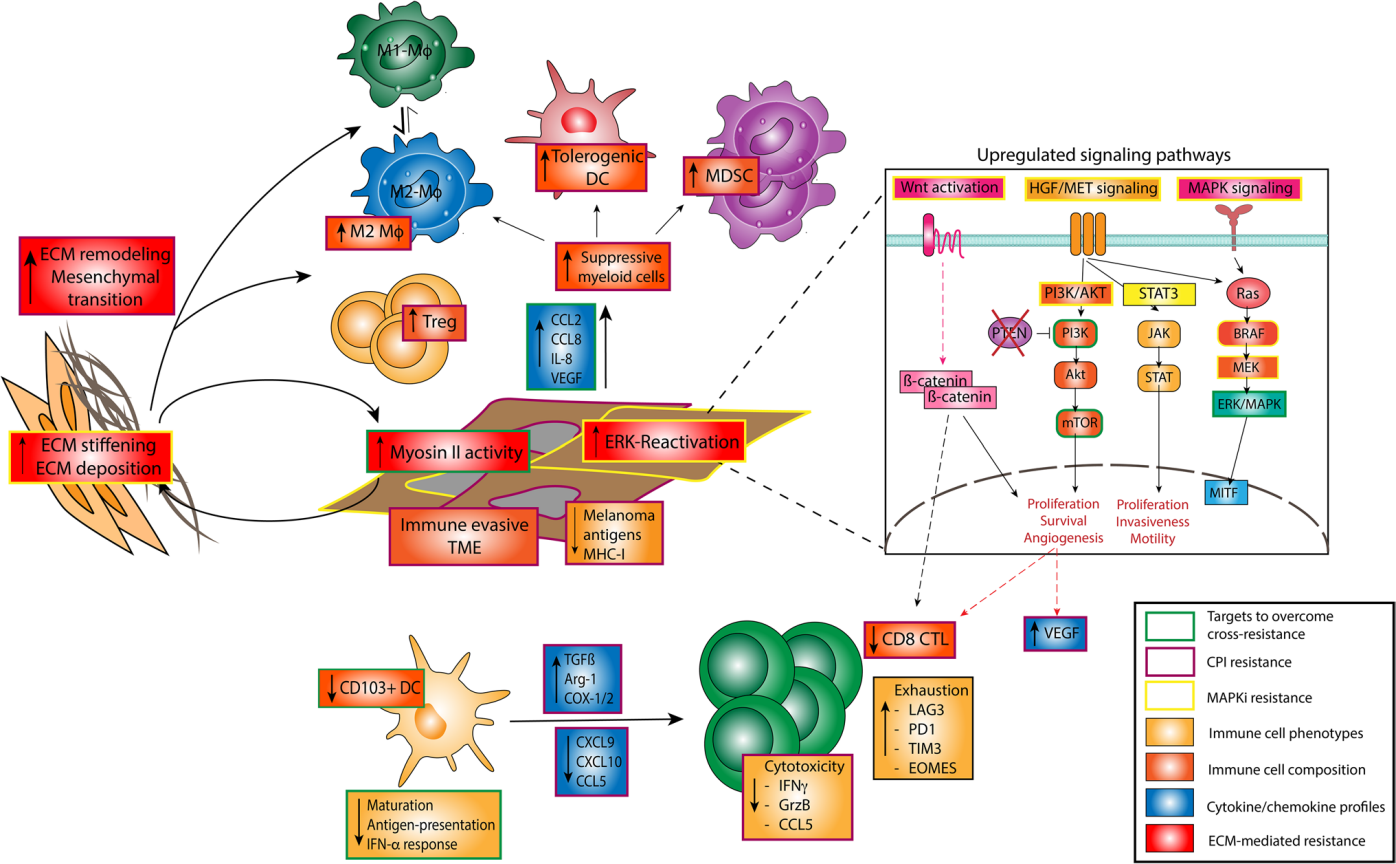
Mechanisms of MAPKi and CPI cross-resistance and potential targets to overcome cross-resistance. Abbreviations: MAPKi = mitogen activated pathway inhibitor(s); TME = tumor microenvironment; DC = dendritic cell; ECM = extracellular matrix; Treg = regulatory T cells; MDSC = myeloid-derived suppressor cells; M1 Mϕ = M1 macrophages; M2 Mϕ = M2 macrophages; CTL = cytotoxic T lymphocytes

As neither triple combination therapy nor sequential treatment regimens of MAPKi and CPI were able to overcome these mechanisms of treatment resistance, there remains a vital need to identify mechanisms of secondary resistance and to develop alternative approaches to re-initiate tumor responses in these patients. In this chapter we will briefly outline the current knowledge on mechanisms of MAPKi and CPI cross-resistance that will eventually help to develop novel treatments to improve the management of advanced melanoma patients (see Fig. 2). In this regard, Hugo and coworkers demonstrated that MAPKi-resistant melanoma showed characteristic non-genomic and immune alterations that also reduced CPI responsiveness [51]. Particularly, the authors reported that the dysregulation of epithelial mesenchymal transition, a downregulation of lymphoid enhancer binding factor 1 (LEF1) transcriptional activity and overexpression of the Yamaguchi sarcoma oncogene-associated Protein 1 (YAP1) reduced melanoma reliance on the MAPK pathway [51]. Further, melanoma tumors of patients showing progressive disease upon MAPKi were characterized by a loss of antigen presentation in most of the tumors, resulting in impaired tumor infiltration of CTL and strong CD8 + T cell exhaustion [51]. Moreover, MAPKi-resistant tumors displayed significantly higher numbers of M2 macrophages that are known to antagonize the recruitment and effector functions of T cells [136]. Transcriptomic analysis of MAPKi-resistant melanoma confirmed the inhibitory role of immunosuppressive myeloid cells as reflected by increased levels of IL-10, VEGF and CCL2 [137]. In addition, these analyses provided evidence that cross-resistance might also be mediated by epithelial-mesenchymal transition of melanoma cells. These early observations are in line with more recent findings by Orgaz and coworkers who demonstrated that treatment-resistant melanoma cells restored Myosin II activity via extracellular matrix (ECM) remodeling to increase survival and thus uncoupling ERK signaling from cytoskeletal remodeling [132]. Notably, the authors further reported that melanoma cells high in Myosin II were secretory and polarized monocytes towards M2 macrophages, while Myosin II activity induced ECM stiffening. To overcome these mechanisms of resistance the authors administered Rho-associated coiled-coil containing protein kinase 1, Myosin II and histone deacetylase inhibitors that resulted in increased melanoma DNA damage, reduced the number of Treg, M2 macrophages and TGF-ß levels and thus enhanced MAPKi and CPI efficacy [132].

The relevance of the immunosuppressive myeloid cell compartment for melanoma cross-resistance was further explored by Steinberg and coworkers who reported that BRAFi resistance was associated with an increasing influx of MDSC into TME that relied on MAPK pathway reactivation and downstream production of myeloid-cell derived CCL2 that could be reversed by the addition of an MDSC blocking C–C chemokine receptor type 2 antagonist [131]. These data have recently been complemented by Haas and coworkers who demonstrated that cross-resistance instructed an immune-evasive TME through enhanced MAPK pathway activity and is characterized by the impaired functionality of CD103^+^ dendritic cells (DC) and an increase in immunosuppressive myeloid cells that could be overcome by direct modulation of the TME via DC maturation and expansion as well as inhibition of the MAPK pathway [28].

Last, Peng and coworkers showed that the loss of the tumor-suppressor PTEN and subsequent activation of the phosphoinositide 3-kinase (PI3K)/protein kinase B pathway promoted immune resistance in melanoma patients via reduction of T cell infiltration of the tumor and an increase of immunosuppressive cytokines such as VEGF that attract tolerogenic DC, MDSC and Treg [133]. Importantly, it has further been reported, that PTEN deficiency did not mediate immune evasion via downregulation of MHC-I or upregulation of PD-L1, but protected tumors from T cell killing through autophagy and by creating an immunosuppressive microenvironment, that could be reversed by a PI3K inhibitor [133]. This non-T-cell inflamed pattern within the TME was further enhanced by activation of the ß-catenin pathway independent from the alterations mediated by PTEN deficiency [133]. In this regard, previous works by Gajewski and coworkers showed that activation of the WNT/ß-catenin signaling pathway contributed to T cell exclusion and resistance to CPI therapy [138].

## Conclusions/Perspectives

The introduction of CPI and MAPK-targeted therapeutics has considerably improved the treatment options for melanoma patients [139, 140]. The mechanisms of action and complementary response kinetics of both treatment modalities and solid preclinical evidence on the favorable effects of upfront MAPKi for anti-tumor immunity supported the hypothesis that the combination of MAPKi and CPI might result in higher rates of rapid and durable response. While results from pivotal phase III trials showed that triple combination therapy, comprising CPI, BRAFi and MEKi, prolonged the duration of response and thereby allowed for long-term PFS, the majority of these trials did not reach their primary endpoint and thus did not support the broad first-line use of triple combination therapies for BRAF^V600^-mutant melanoma patients. Nonetheless, subgroup analyses and preliminary results from trials evaluating triple combination regimens in patients with MBM suggested that patients with higher disease burden and rapidly progressing disease might derive a greater benefit from these treatment regimens. Emerging biomarker analyses might help to better identify this patient population, i.e., it has been reported that particularly patients with a high CD4/CD8 T cell ratio at baseline and a higher circulating tumor (ct)DNA shedding derived a significant benefit from triple combination therapy with spartalizumab, dabrafenib plus trametinib as compared to the DT comparator group [84]. Meanwhile, recently published phase II/III clinical trials evaluating the efficacy of sequential treatment regimens convincingly showed that – in contrast to the previous hypothesis – the upfront use of IPI plus Nivo resulted in significantly better survival outcomes as compared to upfront BRAFi/MEKi treatment, and that the time frame in which to take advantage of upfront BRAFi/MEKi-induced changes of the TME is relatively short [69, 124]. While the relative benefits of upfront combination versus sequential approaches remain a matter of debate, cross-trial comparisons suggest that the upfront use of IPI plus Nivo prior to MAPKi provided at least similar survival rates as compared to triple combination regimens and might therefore be the preferred upfront option for most patients with BRAF^V600^-mutant melanoma. However, prospective clinical trials evaluating triple combination regimens compared to combined checkpoint blockade with IPI plus Nivo as an active comparator are required to adequately address this issue and might particularly delineate their efficacy in specific cohorts of patients including those with more aggressive disease, MBM or those that are refractory towards CPI. Also, these trials might yield more comprehensive data on the efficacy of a short-term run-in of BRAFi/MEKi on the premise of inducing rapid responses before adding CPI in patients with rapidly progressive disease. Regardless of the recent advances in the field of MAPKi/CPI combination therapies an urgent need remains to establish additional treatment options as secondary acquired resistance towards these agents will affect approximately 50% of patients in the long-term and thus poses a vital issue for both treatment options as responses to second-line treatments remain low [25].

## Abbreviations

AE: Adverse event
APC: Antigen presenting cell
BRAF(i): V-raf murine sarcoma viral oncogene homolog B (inhibitor)
CCL2: Chemokine (C–C motif) ligand 2
CPI: Immune-checkpoint inhibitor
ctDNA: Circulating tumor DNA
CTL: Cytotoxic T cell
CTLA-4: Cytotoxic T lymphocyte antigen 4
DC: Dendritic cell
DCR: Disease control rate
DT: Dabrafenib plus Trametinib
ECM: Extracellular matrix
ERK: Extracellular signal regulated kinase
Gp100: Glycoprotein 100
HR: Hazard ratio
DOR: Duration of response
CR: Complete response
NA: Not available
Nivo: Nivolumab
NR: Not reached
ns: Not significant
IFN: Interferon
IL: Interleukin
IPI: Ipilimumab
mAb: Monoclonal antibody
MAPK(i): Mitogen activated protein kinase (inhibitor)
MART-1: Melanoma antigen recognized by T cells
MEK(i): MAPK/ERK Kinase 1 (inhibitor)
ORR: Overall response rate
OS: Overall survival
pCR: Pathological complete response
pRR: Pathological response rate
PD-1: Programmed cell death-protein-1
PD-L1: Programmed cell death-protein ligand 1
PFS: Progression-free survival
PI3K: Phosphoinositide 3-kinase
PTEN: Phosphatase and tensin homolog
TME: Tumor microenvironment
trAE: Treatment-related adverse event
Treg: Regulatory T cell
VEGF: Vascular endothelial growth factor
